# A rare complication of a thoracic wound: the pneumopericardium?

**DOI:** 10.1007/s12024-025-00952-6

**Published:** 2025-01-27

**Authors:** C. Poulain, T. Prigent, B. Guibourg, G. Le Flahec, E. Martin, D. Ben Salem

**Affiliations:** 1 Department of Forensic Medicine, Brest University Hospital, Boulevard Tanguy-Prigent, Brest cedex, 29609 France; 2 Medicolegal private practice, Saint Martin des Champs, France; 3 Departement of Pathology, Brest University Hospital, 2 Avenue Foch, Brest, 29200 France; 4 Medical Information Department, Landerneau Hospital, 1 route de Pencran, Landerneau, 29800 France; 5 LaTIM, Inserm UMR 1101, 22 Avenue Camille-Desmoulins, CS 93837, Brest cedex, 29238 France; 6 Unit of Forensic Imaging, Brest University Hospital, Boulevard Tanguy-Prigent, Brest cedex, 29609 France

**Keywords:** Forensic pathology, Pneumopericardium, Stab wound, Autopsy, Postmortem computed tomography

## Abstract

Pneumopericardium (PPC) is defined by the presence of gas in the pericardial cavity, often leading to cardiac tamponade and a high mortality rate. This report describes a case involving a 33-year-old man found deceased a few meters from a knife, his clothes intact, with no resuscitation attempt made. A knotted scarf was tightly fastened around his neck, without ligature mark. Post-mortem CT revealed 271 ml of gas in the pericardial cavity, with “flattened heart”. The forensic examination revealed two thoracic stab wounds, one penetrated the pericardium without penetrating the heart chamber, while the second remained superficial. In the absence of resuscitation or exsanguination, and in the presence of some non-specific signs observed in the context of asphyxia, the hypothesis of death by compressive PPC was supported. This first French case report of PPC highlights the rarity of this entity in forensic settings, and comparison with other cases described in the literature which did not present the same characteristics points out the diagnostic difficulties it presents and the importance of post-mortem CT in diagnosis.

## Introduction

Pneumopericardium (PPC) is defined by the presence of gas accumulation in the pericardial cavity. PPC was reportedly first described by Bricheteau, a physician at Necker Hospital, in 1844 [1]. It can compress the right heart chambers, resulting in tamponade in some case *(37% according to an remote study)* and a high mortality rate [1–3]. According to Toledo, the causes of PPC can be categorized as follows: iatrogenesis, pericarditis, fistulas between the pericardium and an adjacent air-containing organ, and trauma [4]. Blunt chest trauma is the most frequent traumatic cause of PPC, although thoracic penetrating injury may also lead to its occurrence [1, 5]. Few cases of PPC secondary to stab wounds have been reported in the forensic literature [3, 6].

## Case report

We report the case of a 33-year-old man found deceased in a supine position in a field located in a wetland area. According to the investigation, no resuscitation attempt was undertaken. He was wearing a jacket and a sweatshirt, both open, as well as a white t-shirt, stained with blood on the front, the clothes showing no lesions. He had a history of suicide attempts. A knotted scarf was tightly fastened around his neck, and a Swiss army knife was found approximately 6 m from the body.

**Fig. 1:**
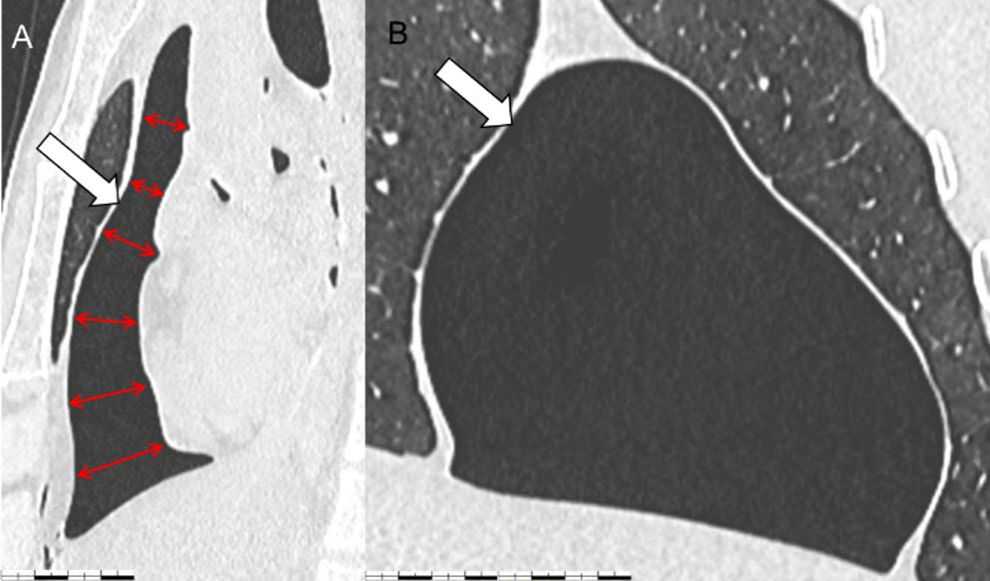
CT scans of the pericardium (sagittal section A and frontal section B) the white arrow indicates the pneumopericardium, while the red double-headed arrows highlight the flattened heart

**Fig. 2:**
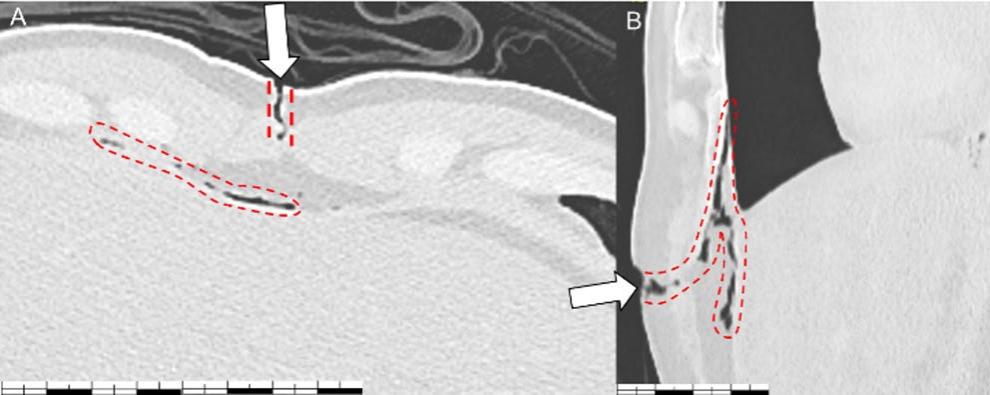
CT scan of the wound (horizontal section A and sagittal section B) white arrow indicates the wound, while the red dotted line highlights the emphysema localized in the subcutaneous soft tissues and mediastinum, particularly anterior to the diaphragm and pericardium

A Post-mortem CT scan showed the presence of a large gaseous collection within the pericardial sac. The pericardial wall appeared bulging and the heart was flattened posteriorly, with compression of the chambers (Figs. 1 and 2). The volume of this collection was radiologically estimated at 271 ml. The Radiological Alteration Index (RA-index) was calculated at 0 (indicating no significant radiological alterations).

During the external body examination conducted by the forensic pathologist on site, conjunctiva and mucosa showed no petechia, bilateral subungual cyanosis and maceration of the extremities were noted. The examination also revealed a very faint circumferential white band around the neck, without any signs of parching (yellow or brown discoloration of the skin). The body showed no signs of decay.

**Fig. 3:**
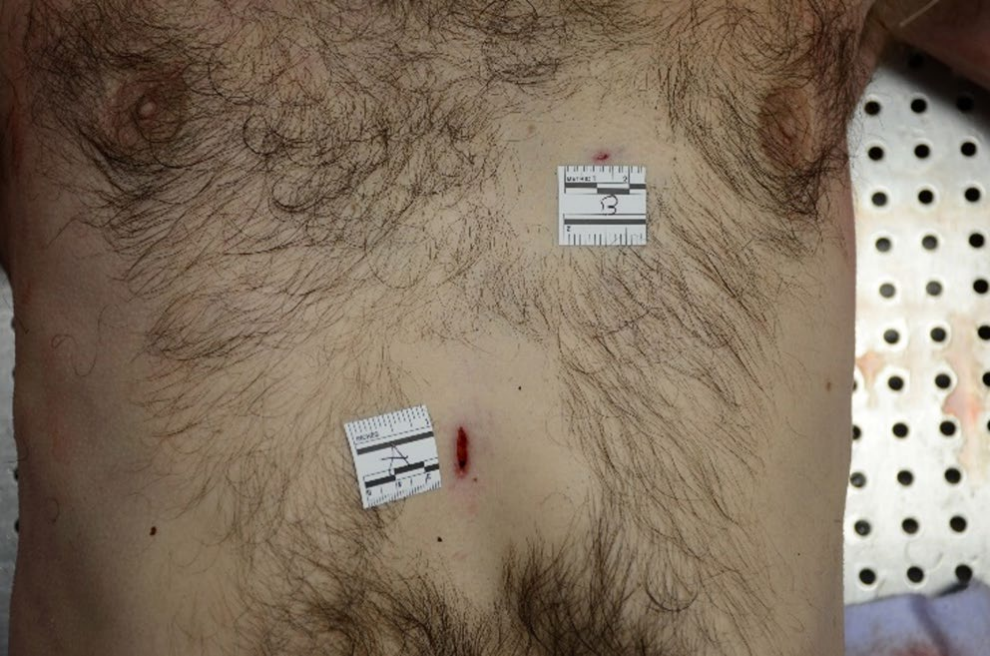
Photographic view of the anterior part of the thorax and the two thoracic wounds

**Fig. 4:**
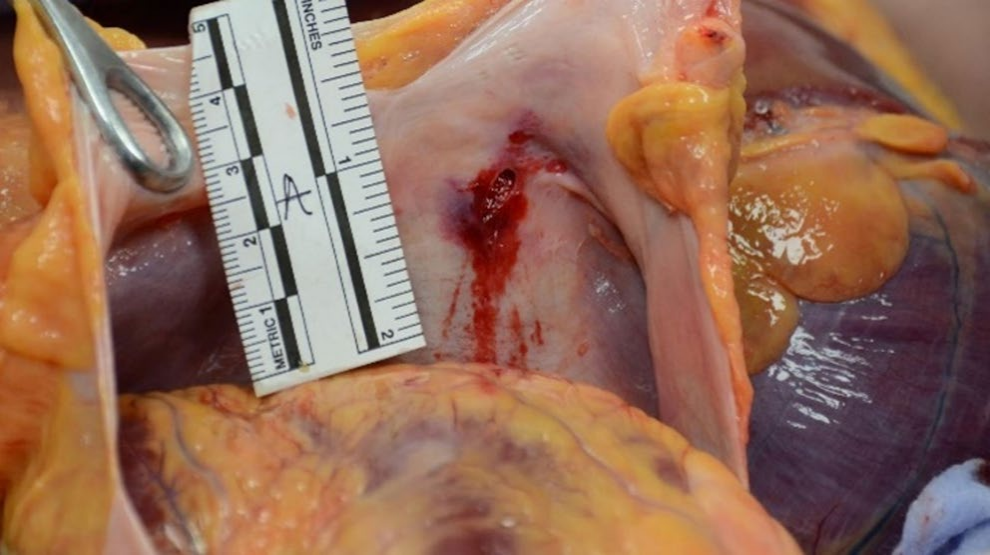
Photographic view of the pericardium showing pericardial wound

**Fig. 5:**
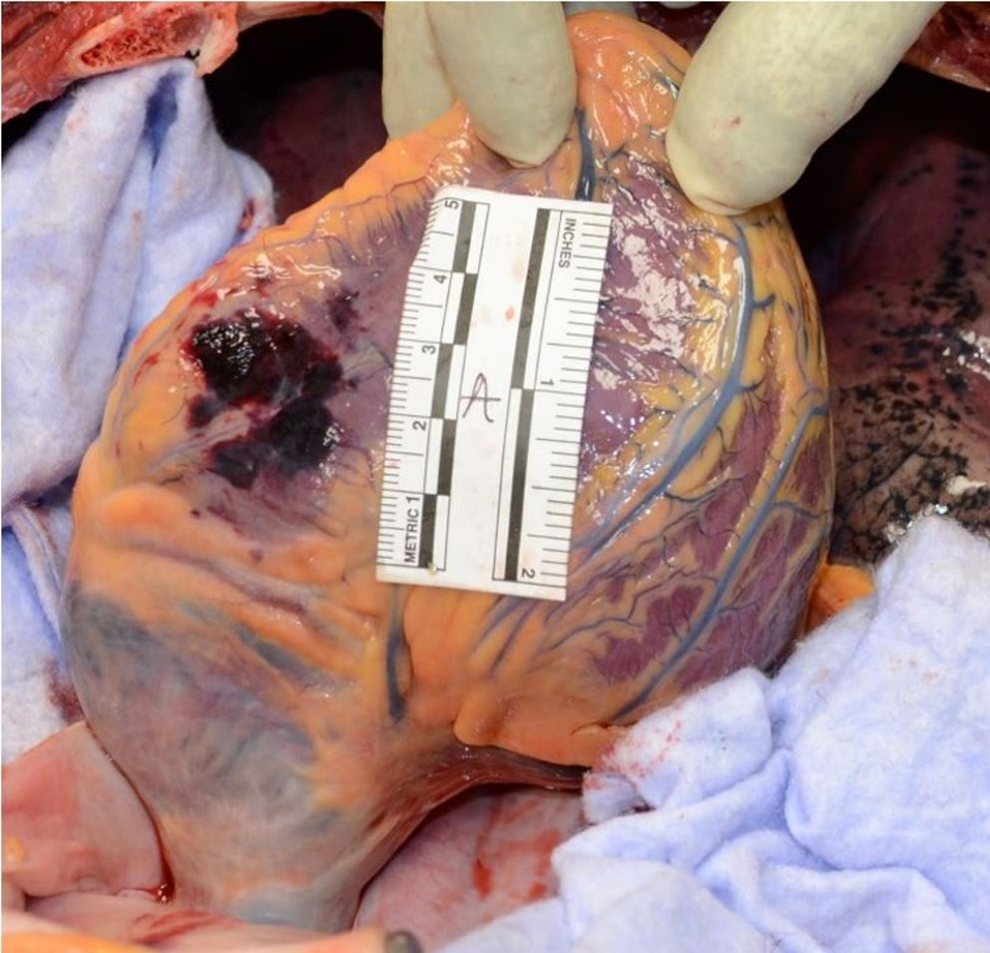
Photographic view of the lower part of the heart showing heart hemorrhagic infiltration

At autopsy, a discrete hemorrhagic infiltration of the subcutaneous fatty tissue, one centimeter in diameter, was observed under the right horizontal branch of the mandible, without cervical muscle infiltration, fracture of the larynx or hyoid bone. Two thoracic wounds were noted, with no corresponding perforation of the clothing. One horizontal wound was located in the left anterior thoracic region, measuring 0.4 cm in length and approximately 0.5 cm in depth. Upon autopsy examination, the wound involved the pectoralis major muscle superficially but did not penetrate into the thoracic cavity (Fig. 3). A second wound, vertical, measuring 1.5 cm in length and approximately 3,6 cm in depth, was located in the subxiphoid region. Its edges were clean, and its upper extremity was sharper (Fig. 3). The autopsy revealed that this wound was penetrating, with a 1 cm injury to the parietal layer of the pericardium at its inferolateral right portion (Fig. 4). There was also a superficial, linear, non-transmural wound, measuring 1 cm on the posterolateral right part of the right ventricle of the heart, associated with hemorrhagic infiltration measuring 3 × 3.5 cm (Fig. 5). Pleural cavities were dry, and the pericardial cavity contained 7 ml of blood. There was no suggestive sign of hemorrhagic shock, with negative findings including: small amount of blood on clothing, visceral congestion, lividity of normal extent. Histological analysis confirmed that the cardiac wound was non-transfixing, it also revealed pulmonary edema and congestion, which are non-specific but can be observed in the context of asphyxia (*indicating an elevation of hydrostatic pressure in the pulmonary circulation*).

In summary, we were in the presence of a non-transfixing cardiac wound with no signs of exsanguination, in the presence of nail cyanosis, a rather congestive body, pulmonary edema and in the absence of petechiae, with a very faint cervical trace without parching. We can therefore conclude, with reasonable confidence, that the cause of death was related to a compressive PPC secondary to a pericardial stab wound. Considering the absence of lesions on the clothing and the notion of a history of suicide attempts, the suicidal hypothesis can be evoked.

## Discussion

PPC is a rare diagnosis in medicine, and even more rarely described in forensic medicine, probably due to the low proportion of traumatic origins in the etiologies described [1–4, 6, 7]. It can be complicated by cardiac tamponade when PPC is under high pressure “tension PPC”, and is called “low-pressure cardiac tamponade” when hemorrhagic shock develops concurently with lower gaz accumultation *(the volume of gas in the pericardial cavity is not as substantial as in high-pressure pneumopericardium however*,* even this smaller volume of gas can contribute to cardiac tamponade*,* especially when combined with hemorrhagic shock*,* leading to lower pressure in cardiac cavity)* [6, 8].

In their case of PPC with tamponade, Heimer et al. showed radiological abnormalities in favour of the compressive nature of the pneumopericardium: the CT scan revealed the so-called “small heart” sign due to compression of the right ventricle, and the pericardium appeared bulging into the right pleural cavity [3]. “Small heart” sign was first described by Mirvis et al. in 1986 during PPC with a decrease in the cardiothoracic index [9]. In 1996, Hernandez-Luyando et al. first described the “flattened heart” sign in PPC, with flattening of the anterior surface of the heart and reduction in its anteroposterior diameter [10].

Diagnosis of PPC is difficult to establish at autopsy, as the air contained in the pericardial sac escapes when it is opened [3, 6]. However, a post-mortem CT scan can reveal it without difficulty, and can provide arguments in favor of tamponade [7, 9–11].

Apart from advanced putrefaction, post-mortem gas production in the pericardial sac is not typical. In our case, there was no evidence of putrefaction, with an RA-Index of 0 [12]. Moreover, given the absence of any reported resuscitation maneuvers and the limited handling of the body after its discovery, iatrogenic origin can be ruled out.

Ro et al. recently reported a case of suicide by stabbing, with two wounds in the epigastric region. The authors argued in favor of a mixed cause of death, secondary to hypovolemia linked to blood depletion following the aortic wound, and to a compressive pneumopericardium (*measured at 133 ml*) [6]. The publication of this article was the subject of an exchange with Zivkovic et al. on the question of whether the pneumopericardium was responsible for the death [13]. Ro et al. argued in favor of this imputability by the fact that thoracic movements, associated with the existence of a check valve function with the liver and diaphragm, would allow the formation of a pneumopericardium. In addition, they pointed out that signs of tamponade such as jugular vein turgor were often absent [14].

Indeed, for a pneumopericardium to become compressive, air must accumulate within the pericardial sac with a one-way valve effect through the wound [1, 3, 15]. During inspiration, the descent of the diaphragm lowers intrathoracic pressure, allowing air to enter the pericardium. During expiration, the upward movement of the diaphragm and liver may compress the wound, reducing or preventing the escape of air [6]. Over successive respiratory cycles, this mechanism could result in a gradual increase in intrapericardial pressure. The resulting pericardial distension may further contribute to this valve effect by mechanically sealing the wound margins. Given the similar anatomical location between our case and previous reports, anatomical factors, such as the proximity of the wound to the diaphragm and interaction with the liver, appear to play a role in creating the valve effect.

Other authors have described cases of compressive pneumopericardium, lethal or not, in association with a stab wound, but this entity remains rare in forensic practice, and the origin of death is sometimes subject to controversy [3, 13–20].

The autopsy case we report is, to our knowledge, the first french publication on the subject. Unlike some of the cases described previously, in our case there is little doubt as to the cause of death. Indeed, there was no hemorrhage likely to explain the death [13]. 

Although the volume of gas accumulated in the pericardial sac may appear significant for suggesting a diagnosis of compressive pneumopericardium (CPP), we believe that the CT scan features such as ‘small heart’ and ‘flattened heart’ are more relevant, as they directly reflect the pressure exerted by the pneumopericardium on the heart. Conversely, the gas volume may vary depending on the individual’s morphology and anatomical particularities, making it a less reliable indicator of clinical impact. In our case, the presence of signs of congestion and a compressed pneumopericardium (flattened heart on PMCT) strongly suggests that the elevated intrapericardial pressure caused significant cardiac compression. This likely impaired diastolic filling, particularly of the right ventricle, reducing cardiac output and leading to acute heart failure. The resulting increase in hydrostatic pressure within the pulmonary vessels explains the observed pulmonary congestion and edema, supporting cardiogenic shock as the probable cause of death. Additionally, the small wound and hemorrhagic infiltration on the heart’s surface could have directly irritated the myocardium, potentially triggering a rhythm or conduction disorder that contributed to the fatal outcome.

Keypoints: 

Post-mortem computed tomography is crucial for diagnosing and characterizing PPC, particularly in visualizing its compressive effects, such as a'flattened' or'small' heart, which aid in determining the mechanism of death.

While essential for diagnosing PPC, post-mortem imaging remains a complementary tool. Autopsy remains indispensable for accurately determining the cause of death through comprehensive macroscopic findings and further investigations.

## Data Availability

N/A.
